# Additive Regulation of Adiponectin Expression by the Mediterranean Diet Olive Oil Components Oleic Acid and Hydroxytyrosol in Human Adipocytes

**DOI:** 10.1371/journal.pone.0128218

**Published:** 2015-06-01

**Authors:** Egeria Scoditti, Marika Massaro, Maria Annunziata Carluccio, Mariangela Pellegrino, Martin Wabitsch, Nadia Calabriso, Carlo Storelli, Raffaele De Caterina

**Affiliations:** 1 National Research Council (CNR) Institute of Clinical Physiology, Lecce, Italy; 2 Department of Biological and Environmental Science and Technology (DISTEBA), University of Salento, Lecce, Italy; 3 Division of Pediatric Endocrinology, Diabetes and Obesity, Department of Pediatrics and Adolescent Medicine, University of Ulm, Ulm, Germany; 4 “G. d’Annunzio” University and Center of Excellence on Aging, Chieti, Italy; 5 “G. Monasterio” Foundation for Clinical Research, Pisa, Italy; Institute of Hepatology - Birkbeck, University of London, UNITED KINGDOM

## Abstract

Adiponectin, an adipocyte-derived insulin-sensitizing and anti-inflammatory hormone, is suppressed in obesity through mechanisms involving chronic inflammation and oxidative stress. Olive oil consumption is associated with beneficial cardiometabolic actions, with possible contributions from the antioxidant phenol hydroxytyrosol (HT) and the monounsaturated fatty acid oleic acid (OA, 18:1n-9 *cis*), both possessing anti-inflammatory and vasculo-protective properties. We determined the effects of HT and OA, alone and in combination, on adiponectin expression in human and murine adipocytes under pro-inflammatory conditions induced by the cytokine tumor necrosis factor(TNF)-α. We used human Simpson-Golabi-Behmel syndrome (SGBS) adipocytes and murine 3T3-L1 adipocytes as cell model systems, and pretreated them with 1-100 μmol/L OA, 0.1-20 μmol/L HT or OA plus HT combination before stimulation with 10 ng/mL TNF-α. OA or HT significantly (P<0.05) prevented TNF-α-induced suppression of total adiponectin secretion (by 42% compared with TNF-α alone) as well as mRNA levels (by 30% compared with TNF-α alone). HT and OA also prevented—by 35%—TNF-α-induced downregulation of peroxisome proliferator-activated receptor PPARγ. Co-treatment with HT and OA restored adiponectin and PPARγ expression in an additive manner compared with single treatments. Exploring the activation of JNK, which is crucial for both adiponectin and PPARγ suppression by TNF-α, we found that HT and OA additively attenuated TNF-α-stimulated JNK phosphorylation (up to 55% inhibition). In conclusion, the virgin olive oil components OA and HT, at nutritionally relevant concentrations, have additive effects in preventing adiponectin downregulation in inflamed adipocytes through an attenuation of JNK-mediated PPARγ suppression.

## Background

Obesity is associated with an increased risk for cardiometabolic complications, including type 2 diabetes (T2DM) and cardiovascular disease (CVD), two leading causes of morbidity and mortality [1,2]. Although pathophysiological mechanisms linking obesity to cardiometabolic disturbances are incompletely understood, compelling evidence suggests that obesity is characterized by an aberrant production of adipokines, among which tumor necrosis factor(TNF)-α, derived from both adipocytes and infiltrating macrophages, as well as by a state of tissue and systemic chronic, low-grade inflammation and increased oxidative stress, which likely play a key role in both T2DM and CVD [1,2].

Adiponectin is a major adipocyte-secreted adipokine, abundantly present in the circulation of healthy humans and mice and exerting anti-diabetic, anti-inflammatory and anti-atherosclerotic effects [3]. Unlike most other adipokines, local and circulating levels of adiponectin decrease in obesity and related conditions, including insulin resistance, T2DM, endothelial dysfunction, hypertension, and atherosclerosis, all contributing to CVD in general and coronary heart disease in particular [3]. Genetic variations associated with low plasma adiponectin levels predispose to insulin resistance and CVD, and increased circulating adiponectin—by either genetic or pharmacological approaches—has been shown to ameliorate insulin sensitivity in the liver and the skeletal muscle, as well as glucose tolerance [3]. Therefore, pharmacological and/or dietary strategies able to restore adiponectin expression and secretion by improving inflammation-associated adipocyte dysfunction might beneficially impact several obesity-related metabolic and cardiovascular complications. Interestingly, in addition to adiponectin alone, the related leptin to adiponectin ratio has been recently emerged as a reliable and clinically useful marker of cardiometabolic risk, as well as a valuable indicator of the effectiveness of anti-diabetic therapy [4].

Over the past several years, Mediterranean diets rich in virgin olive oil have received much attention for health benefits, including a lower morbidity and mortality from CVD [5], and a lower incidence of obesity and of the metabolic syndrome [6] compared with other patterns of food intake. Mediterranean dietary patterns have been positively and independently associated with total adiponectin plasma levels in diabetic [7], obese women [8], in patients at high CV risk [9] as well as in healthy populations [10]. These beneficial effects may be at least partially ascribed to the consumption of virgin olive oil, a functional food rich in antioxidants, including the ortho-diphenolic compound hydroxytyrosol (2-[3,4-dihydroxyphenil]-ethanol, HT) as a main component, and characterized by a favorable fatty acid (FA) profile, with a high content of the monounsaturated fatty acid (MUFA) oleic acid (OA, 18:1n-9, *cis*) [11]. HT has potent anti-inflammatory, anti-thrombotic, and anti-atherogenic properties, improving endothelial dysfunction, haemostatic and lipid profiles, and decreasing oxidative stress and inflammatory cell activation [12–15]. Recently, beneficial HT effects against hyperglycemia, insulin resistance and the metabolic syndrome have been reported [16,17]. On the other hand, also OA or OA-rich diets have been reported to exert a number of anti-inflammatory and vasculo-protective activities *in vitro* and *in vivo* and to provide improvements in insulin resistance and T2DM [18].

We therefore hypothesized that HT and OA, alone and possibly in concert, might improve adipocyte dysfunction by stimulating adiponectin expression as an additional mechanism for their beneficial metabolic and anti-inflammatory action. We here therefore evaluated the effects of HT and OA on the TNF-α-induced downregulation of adiponectin expression in adipocytes, and explored underlying mechanisms.

## Materials and Methods

### Materials

HT (≥98% purity), the peroxisome proliferator-activated receptor (PPAR)γ agonist rosiglitazone (RSG), and the PPARγ antagonist GW9662 were obtained from Cayman Chemicals (Ann Arbor, MI, USA). Recombinant human insulin was from Roche Diagnostics (Mannheim, Germany). All other chemicals, including sodium OA and TNF-α, were obtained from Sigma Aldrich (St. Louis, MO, USA), unless otherwise indicated. OA was conjugated with fatty acid-free bovine serum albumin at a 4:1 molar ratio.

### Cell cultures and treatments

In the current study, as human adipose tissue material with potential to differentiate is limited, we used human Simpson-Golabi-Behmel syndrome (SGBS) preadipocytes, a physiologically relevant cell model system resembling human adipose tissue [19]. These, originally derived from the stromal fraction of subcutaneous adipose tissue of an infant with the Simpson-Golabi-Behmel syndrome, were a generous gift of Prof. Martin Wabitsch (Division of Pediatric Endocrinology, Diabetes and Obesity, Department of Pediatrics and Adolescent Medicine, University of Ulm, Ulm, Germany, among the study co-authors). SGBS cells are characterized by a high capacity for adipogenic differentiation over many generations, and functionally behave like human primary adipocytes [19]. SGBS preadipocytes were cultured and differentiated into mature adipocytes as previously described [19]. Briefly, SGBS cells were maintained in Dulbecco's Modified Eagle Medium (DMEM)/F12 containing 10% fetal bovine serum (FBS) and 1% penicillin/streptomycin, 33 μmol/L biotin and 17 μmol/L pantothenate. For experimental purposes, cells were plated and allowed to reach confluence before the addition of serum-free differentiation medium [Dulbecco's Modified Eagle Medium: Nutrient Mixture F-12 (DMEM/F12) with 25 nmol/L dexamethasone, 250 μmol/L 3-isobutyl-1-methylxanthine, 2 μmol/L RSG, 0.01 mg/mL human transferrin, 20 nmol/L insulin, 100 nmol/L cortisol, 0.2 nmol/L triiodothyronine, 33 μmol/L biotin, and 17 μmol/L pantothenate] for 4 days. Cell medium was then changed to an adipogenic medium (DMEM/F12 with 0.01 mg/mL human transferrin, 20 nmol/L insulin, 100 nmol/L cortisol, 0.2 nmol/L triiodothyronine, biotin, and pantothenate) for further 10 days. On day 15, >90% of these cells undergo complete differentiation into mature adipocytes, as assessed using Oil Red-O lipid staining and the expression of adipocyte-specific mRNAs, such as lipoprotein lipase, adipocyte fatty acid binding protein (FABP4), peroxisome proliferator-activated receptor(PPAR)-γ, and the glucose transporter GLUT-4.

We also used 3T3-L1 mouse embryo fibroblasts as an additional model system of fat cells. These were obtained from American Type Culture Collection (ATCC) (Manassas, VA, USA) and cultured in DMEM containing 10% bovine calf serum until confluent. Two days after confluence (day 0), cells were stimulated to differentiate into adipocytes with DMEM containing 10% FBS, 1 μg/mL insulin, 1 μmol/L dexamethasone, and 0.5 mmol/L 3-isobutyl-1-methylxanthine for 2 days. Cells were then maintained in 10% FBS/DMEM medium with 1 μg/mL insulin for additional 2 days, followed by culturing with 10% FBS/DMEM medium until the analysis. Preliminary studies showed that 10 days after induction of differentiation, >90% of cells displayed the characteristic lipid-filled adipocyte phenotype.

Local inflammation in adipose tissue was mimicked by incubating fully differentiated SGBS cells or 3T3-L1 adipocytes with medium supplemented with the pro-inflammatory cytokine TNF-α at 10 ng/mL during 24 h. Unstimulated controls were adipocytes incubated in medium without TNF-α. A 24-h treatment with 10 ng/mL TNF-α was chosen on the basis of a pilot dose- and time-course study, showing maximal reduction of both adiponectin protein and mRNA levels after 10 ng/mL TNF-α for 24 h, in the absence of any effect on cell viability (data not shown). For HT and OA treatments, SGBS cells or 3T3-L1 adipocytes were incubated with 1–100 μmol/L OA for 48 h or with 0.1–20 μmol/L HT for 1 h, alone or in combination, before stimulation with 10 ng/mL TNF-α. Our preliminary analysis of the fatty acid composition of cellular total lipids revealed that 48-h cell pretreatment with OA was associated with an increase in the amount of OA compared with untreated control (data not shown). A pretreatment time of 1 h was chosen for HT in consideration of the pharmacokinetics of plasma HT after virgin olive oil intake, with a time to reach the peak concentration of 1 h and an elimination half-life of 2–3 h [20]. In some experiments, mitogen-activated protein kinase (MAPK) inhibitors were added 1 h before TNF-α.

### Cell viability

Cell viability was determined by the 3-(4,5-dimethylthiazol-2-yl)-2,5-diphenyl tetrazolium bromide (MTT) assay, a commonly used method to evaluate cell survival, based on the ability of viable cells to convert MTT, a soluble tetrazolium salt, into an insoluble formazan precipitate, which is quantitated spectrophotometrically. Briefly, after the pertinent treatment, cells were incubated with MTT (0.5 mg/mL) for 4 h, and the formazan products were then dissolved by isopropanol. Absorbance was measured at 490 nm on a microplate reader.

### Measurement of total adiponectin in culture media

Media were collected 24 h after TNF-α treatment, centrifuged for 5 min, and stored at -20°C until analysis. Levels of adiponectin in the culture medium were determined using a Quantikine Human or Mouse Adiponectin/Acrp 30 ELISA kit (R&D Systems, Minneapolis, MN, USA) according to the manufacturer’s instructions. Adiponectin concentration was calculated from the standard curve, normalized to total protein content, and expressed as percent of unstimulated control.

### Measurement of leptin in culture media

Levels of leptin in the culture medium were determined using a Quantikine Human Leptin ELISA Kit (R&D Systems) according to the manufacturer’s instructions. Leptin concentration was calculated from the standard curve, normalized to total protein content, and expressed as percent of unstimulated control.

### Immunocytochemistry

3T3-L1 adipocytes grown in 24-well plates on coverslips (Thermanox, ProSciTech, Thuringowa, Queensland, Australia) were pretreated with HT or OA before TNF-α stimulation for 24 h. The specimens were fixed with cold acetone and then incubated overnight with a rabbit polyclonal antibody against adiponectin (Millipore, Billerica, MA, USA). After 3 washes with PBS, the specimens were incubated for 1 h with a biotinylated anti-rabbit IgG antibody (Santa Cruz Biotechnology, Santa Cruz, CA, USA) and for 1 h with extravidin peroxidase (Sigma). After the incubation with diaminobenzidine (Sigma) for 30 min, coverslips with the stained cells were mounted and photographed (x40 magnification).

### Cell lysis and Western blotting

After treatments, whole cell lysates were prepared by the addition of a lysis buffer (150 mmol/L (4-(2-hydroxyethyl)-1-piperazineethanesulfonic acid) (HEPES), pH 7.9, 150 mmol/L NaCl, 1 mmol/L ethylenediaminetetraacetic acid (EDTA), 1% Triton-X100, 10% glycerol, 1 mmol/L diethyldithiocarbamate, 1 mmol/L phenylmethylsulfonyl fluoride, 1 mg/mL aprotinin, 1 mg/mL leupeptin). Lysates were incubated on ice for 30 min, and then centrifuged at 10,000 × g for 20 min at 4°C. Equal amounts of cell protein samples (20 μg) were separated using NuPAGE Bis-Tris precast 10% polyacrylamide gels under reducing condition (Invitrogen, Carlsbad, CA, USA). Resolved proteins were transferred onto nitrocellulose sheets (Amersham, Freiburg, Germany), and the resulting membranes were saturated with 5% blocking agent (Amersham) in Tris-buffered saline (TBS, 20 mmol/L Tris, pH 7.6, 132 mmol/L NaCl)/0.1% Tween 20 for 1 h at room temperature. Blots were then incubated overnight at 4°C with primary antibodies against adiponectin (Millipore), PPARγ, c-Jun N-terminal kinase (JNK)1, JNK2, phospho-JNK (pJNK) (Thr183/Tyr185) (Santa Cruz), total JNK (Millipore), and β-actin (Sigma), followed by a horseradish peroxidase-conjugated secondary antibody (Santa Cruz). An enhanced chemiluminescence (ECL) kit (Amersham) was used to reveal positive bands, according to manufacturer’s instructions. Bands were analyzed quantitatively using the Scion Image Alpha 4.0.3.2 software (Scion Corporation) and normalized to β-actin levels.

### RNA isolation and real-time quantitative polymerase chain reaction

Total RNA was isolated by using the TRIzol reagent (Invitrogen) according to the manufacturer’s protocol. For real-time quantitative polymerase chain reaction (qPCR), total RNA (2 μg) was converted into first-strand cDNA by using the High Capacity cDNA Reverse Transcription Kit (Applied Biosystems, Monza, Italy). The qPCR was performed in an Applied Biosystems 7500 FAST Real Time PCR System by using Taqman Gene Expression Assays for adiponectin, PPARγ, JNK1 and JNK2. All reactions were done in triplicate, and the amount of mRNA was calculated by the comparative critical threshold (C_T_) method. To account for possible variations related to cDNA input or the presence of PCR inhibitors, the endogenous reference gene ribosomal 18 S was simultaneously quantified for each sample, and the data normalized accordingly. Results are expressed as fold increase relative to unstimulated control (= 1).

### Preparation of nuclear extracts and measurement of PPARγ DNA binding activity

Cells were rinsed with ice-cold PBS containing phosphatase inhibitors (pH 7.4), scraped, and collected by centrifugation at 300 × g for 5 min at 4°C. The pellet was then incubated for 30 min in ice-cold hypotonic buffer (20 mmol/L HEPES, 5 mmol/L NaF, 10 μmol/L Na_2_MoO_4_, 0.1 mmol/L EDTA, 0.5% Nonidet P-40; pH 7.5) and centrifuged at 8000 ×g for 5 min at 4°C. The nuclear pellet was resuspended in ice-cold lysis buffer (20 mmol/L HEPES pH 7.5, 0.35 mol/L NaCl, 20% glycerol, 1% Nonidet P-40, 1 mmol/L MgCl_2_•6H_2_O, 0.5 mmol/L EDTA, 0.1 mmol/L ethylene glycol tetraacetic acid (EGTA), 5 mmol/L dithiothreitol (DTT)) containing a protease inhibitor cocktail (Sigma). After 30 min of incubation at 4°C, the lysate was centrifuged for 10 min at 15,000 × g and the supernatant stored at -80°C. For measurement of PPARγ DNA binding activity, a Transcription Factor Assay ELISA kit (Cayman Chemicals, Ann Arbor, MI, USA) was used according to the manufacturer’s instruction. Briefly, nuclear proteins (10 μg) were incubated overnight at 4°C with an oligonucleotide containing a PPARγ response element, which was immobilized onto a 96-well plate in the presence of competitive binding with the wild-type or mutated consensus oligonucleotide. After washing, an anti-PPARγ antibody was added into each well and further incubated for 1 h at 25°C. The wells were washed again and then incubated with peroxidase-conjugated secondary antibody for 1 h at 25°C. After addition of the developing solution, the optical density was read at 450 nm. Data are expressed as percent of unstimulated control.

### Targeting silencing of JNK by small interfering RNA

Validated small interfering RNAs (siRNA) against JNK1 or JNK2 and a scrambled negative control siRNA were obtained from Qiagen (Milan, Italy). Two different siRNA sequences were used for targeted silencing of each JNK isoform. SGBS cells were transfected with 45 nmol/L control siRNA, JNK1 and/or JNK2 siRNA using the Lipofectamine RNAi/MAX reagent (Invitrogen) according to the manufacturer’s instructions. Knockdown of JNK mRNA and protein was assessed after 72 h post-transfection by qPCR using Taqman gene expression assays for JNK1 and JNK2, and by Western blotting using antibodies against JNK1 and JNK2. After 72 h of transfection, the medium was changed, and cells were stimulated with or without 10 ng/mL TNF-α for 24 h. Adiponectin release in the culture medium and intracellular protein levels were measured by ELISA and Western analysis, respectively, while adiponectin mRNA levels were determined by qPCR.

### Statistical analysis

Results are expressed as means ± SD of at least 3 independent experiments performed in triplicate. We used the Student’s t test for comparing means between control group and compound-treated group. We performed multiple comparisons by one-way analysis of variance (ANOVA). A *p* level <0.05 was considered statistically significant.

## Results

### HT and OA additively attenuate TNF-α-mediated decrease in adiponectin expression in adipocytes

**Fig 1:**
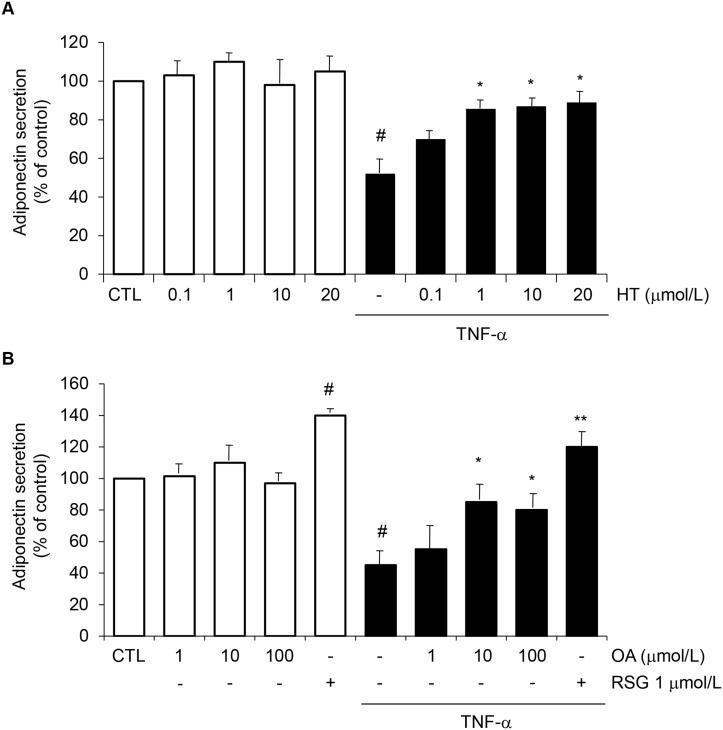
Attenuation by HT and OA of TNF-α-induced inhibition of adiponectin protein release in human adipocytes. Human SGBS adipocytes were pretreated with HT (1 h) (A), OA (48 h) or RSG (24 h) (B) at the concentrations indicated and then either treated with 10 ng/mL TNF-α (black-filled bars), or left untreated (open white bars), for 24 h. Adiponectin levels in the culture medium were determined by ELISA, and expressed as percent of unstimulated control (CTL). Bars represent means ± SD (n = 3). #*p*<0.05 versus CTL. *p<0.05 versus TNF-α. **p<0.01 versus TNF-α.

**Fig 2:**
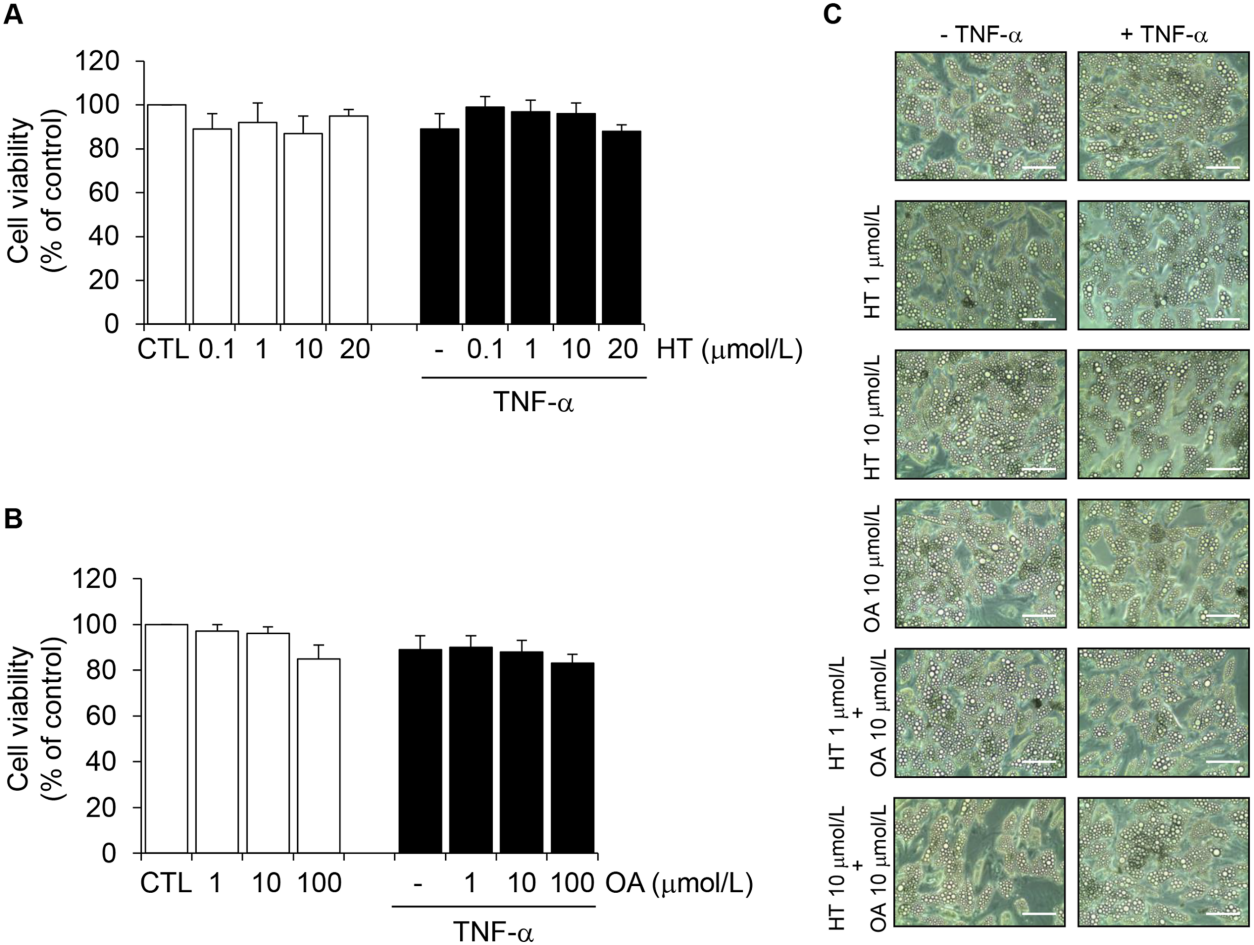
The effect of HT and OA treatment on cell viability. SGBS adipocytes were treated with HT (1 h) (A) or OA (48 h) (B) at the concentrations indicated, and then either treated with 10 ng/mL TNF-α (black-filled bars), or left untreated (open white bars), for 24 h. Cell viability was assessed by the MTT assay, and expressed as percent of unstimulated control (CTL). Data are means ± SD (n = 3). In (C), phase-contrast images of adipocytes treated with HT, OA or HT + OA in the absence or presence of TNF-α are presented. Scale bar = 50 μm.

We studied the effects of HT (0.1–20 μmol/L) and OA (1–100 μmol/L) separately on total adiponectin protein levels in the culture medium of SGBS cells. Cell exposure to TNF-α for 24 h resulted in a significant reduction of adiponectin protein levels in the culture medium, as assessed by ELISA (Fig 1). HT concentration-dependently prevented adiponectin decrease in response to TNF-α, with a significant effect starting at 1 μmol/L (30% ± 5% reversal compared with TNF-α alone) (Fig 1A). At ≥ 10 μmol/L OA was also effective in attenuating TNF-α-induced adiponectin downregulation (Fig 1B). As a positive control we used the thiazolidinedione (TZD) and PPARγ agonist RSG, which resulted in a significant increase of adiponectin secretion in the absence or presence of TNF-α (Fig 1B), in agreement with earlier studies [21]. Since in the absence of TNF-α neither HT nor OA significantly influenced adiponectin levels (Fig 1, white bars), we did not further pursue the characterization of HT or OA effects on basal adiponectin expression. Treatment of SGBS cells with HT or OA in the absence or presence of TNF-α, at the concentrations and times used in our assays, did not affect cell viability, as assessed by the MTT test (Fig 2A and 2B), morphological observation (Fig 2C), protein assay and Trypan blue exclusion (data not shown).

**Fig 3:**
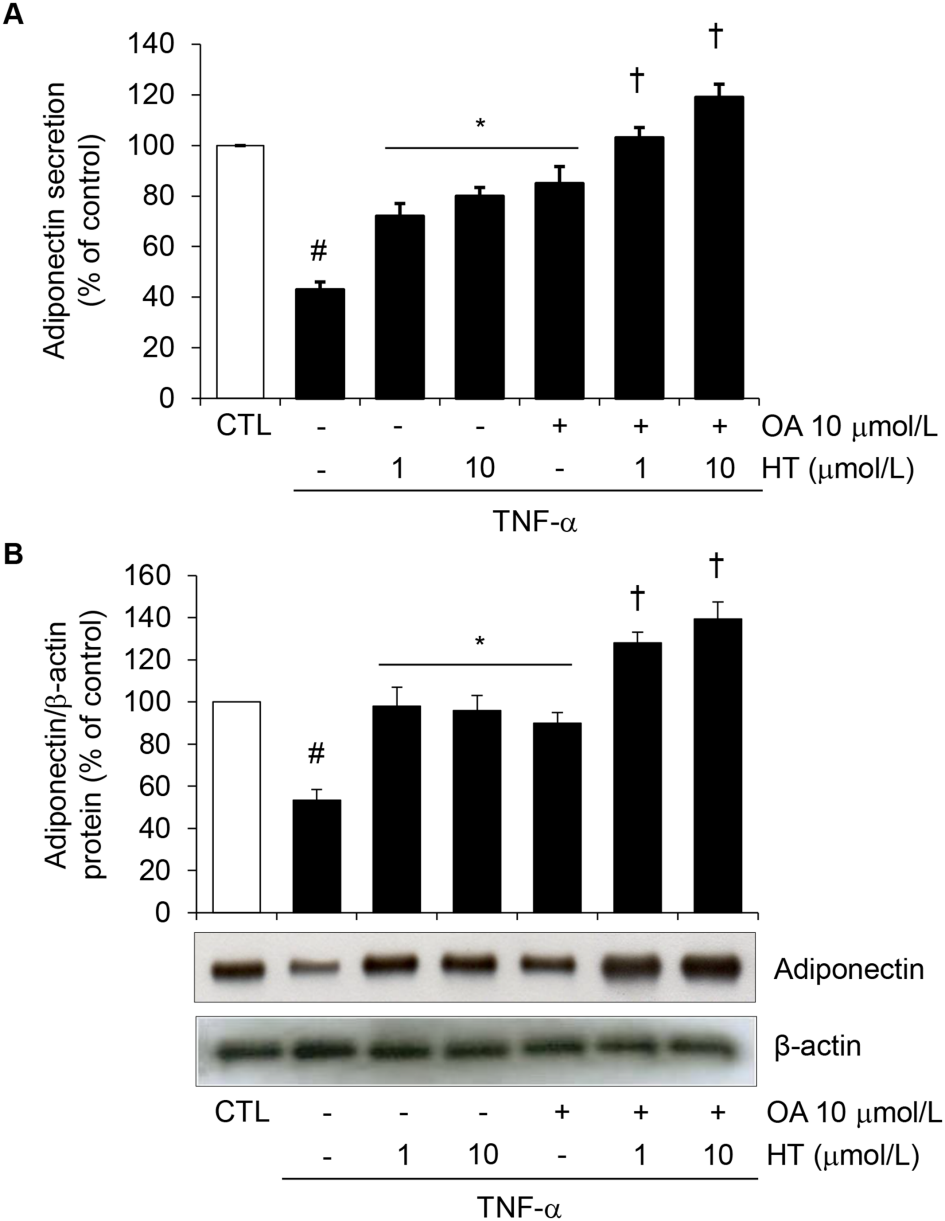
Effect of combined treatment with HT and OA on TNF-α-induced inhibition of adiponectin protein release and expression. SGBS cells were pretreated with either HT, OA or cotreated with HT + OA before 10 ng/mL TNF-α stimulation for 24 h. (A) Adiponectin in the culture medium was determined by ELISA, and expressed as percent of unstimulated control (CTL). (B) Adiponectin intracellular protein levels were determined by Western analysis using antibodies against adiponectin. Western analysis under reducing and denaturing condition here reveals the 30 kDa adiponectin monomer. Adiponectin expression was normalized to β-actin, and expressed as percent of unstimulated control (CTL). Data are means ± SD (n = 3). #*p*<0.05 versus CTL. *p<0.05 versus TNF-α alone. †*p*<0.05 versus each compound + TNF-α.

**Fig 4:**
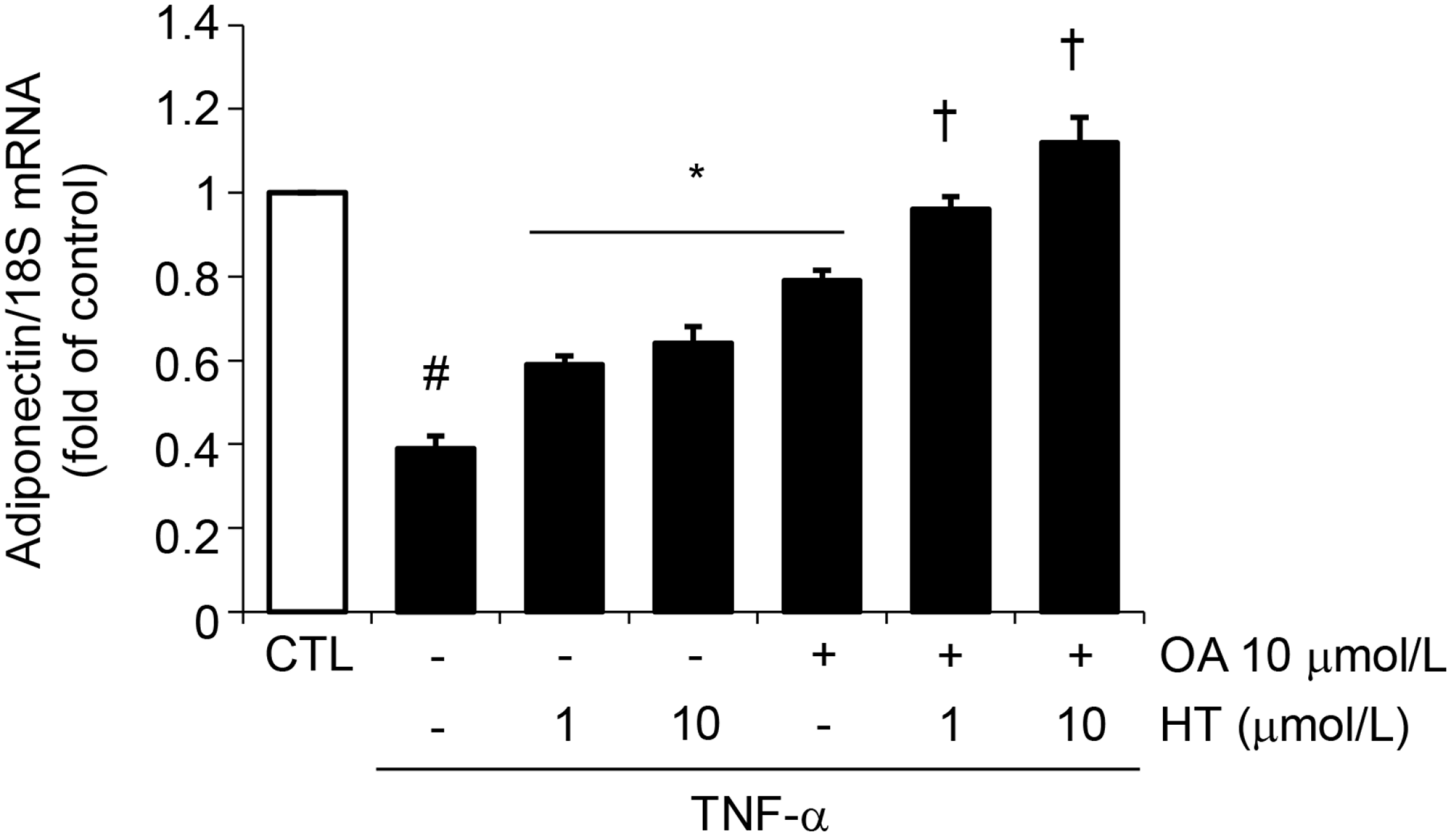
Effect of combined treatment with HT and OA on TNF-α-induced inhibition of adiponectin mRNA expression. SGBS cells were pretreated with either HT, OA or cotreated with HT + OA before 10 ng/mL TNF-α stimulation for 24 h. Adiponectin mRNA levels were determined by qPCR and normalized to 18S RNA. Data are expressed as fold induction over unstimulated control (CTL). Data are means ± SD (n = 3). #*p*<0.05 versus CTL. *p<0.05 versus TNF-α alone. †*p*<0.05 versus each compound + TNF-α.

Cell treatment with a combination of physiologically relevant concentrations of OA (10 μmol/L) plus HT (1–10 μmol/L) before TNF-α restored intracellular and secreted protein levels (Fig 3A and 3B) as well as mRNA levels (Fig 4) of adiponectin in an additive manner compared with single treatments, thus suggesting an important additive effects of these two compounds simultaneously present in virgin olive oil.

To extend the observation made in SGBS adipocytes to other adipocyte-like cells, differentiated murine 3T3-L1 adipocytes were also used. As in SGBS cells, HT and OA, alone and—additively in combination—restored adiponectin release in the culture medium (S1 and S2 Figs), as well as intracellular protein and mRNA levels (S2 Fig), with the exception of OA, needing higher concentrations (≥ 100 μmol/L) to be effective in counteracting TNF-α-induced adiponectin reduction. The subsequent experiments were performed in SGBS cells, although validation of each essential findings was done with 3T3-L1 adipocytes.

To further confirm and expand findings of HT and OA modulating effect on adipocyte dysfunction, we analyzed whether HT and OA—besides improving adiponectin—affected the production of leptin, another key adipokine with metabolic and vascular effects opposite to those of adiponectin; and of the leptin-to-adiponectin ratio, a recently recognized predictor of adipocyte dysfunction-related cardiometabolic risk [4]. To this aim, the release of leptin in the culture medium and the leptin-to-adiponectin ratio were measured in SGBS adipocytes after HT or OA treatment in the absence or presence of TNF-α. As shown in S3 Fig, HT, at all the concentrations tested (0.1–10 μmol/L), and OA at 10 and 100 μmol/L significantly decreased leptin release compared with untreated control. The combination of HT and OA did not here result in any significant additive effect compared with single treatments (not shown). TNF-α treatment for 24 h at 10 ng/mL reduced leptin secretion by about 40% compared with unstimulated control, but neither HT nor OA alone or in combination (not shown) affected leptin levels in response to TNF-α.

As a consequence of the reducing effect on leptin and of the neutral effects on adiponectin in basal unstimulated control, the leptin-to-adiponectin ratio significantly decreased, by about 25 ± 3%, in the presence of HT at all the concentrations tested or of OA at 10 and 100 μmol/L compared with untreated control (S3 Fig). TNF-α did not significantly change the leptin-to-adiponectin ratio compared with unstimulated control, but both HT and OA significantly decreased the leptin-to-adiponectin ratio (by 38 ± 4%) compared with TNF-α alone, and this occurred in an additive manner for OA plus HT treatment (S3 Fig). These results suggested that adiponectin restoration by HT and OA is accompanied by the reduction of leptin release and, more importantly, the concomitant improvement of the leptin-to-adiponectin ratio.

### HT and OA additively prevent TNF-α-induced inhibition of PPARγ expression and binding activity

**Fig 5:**
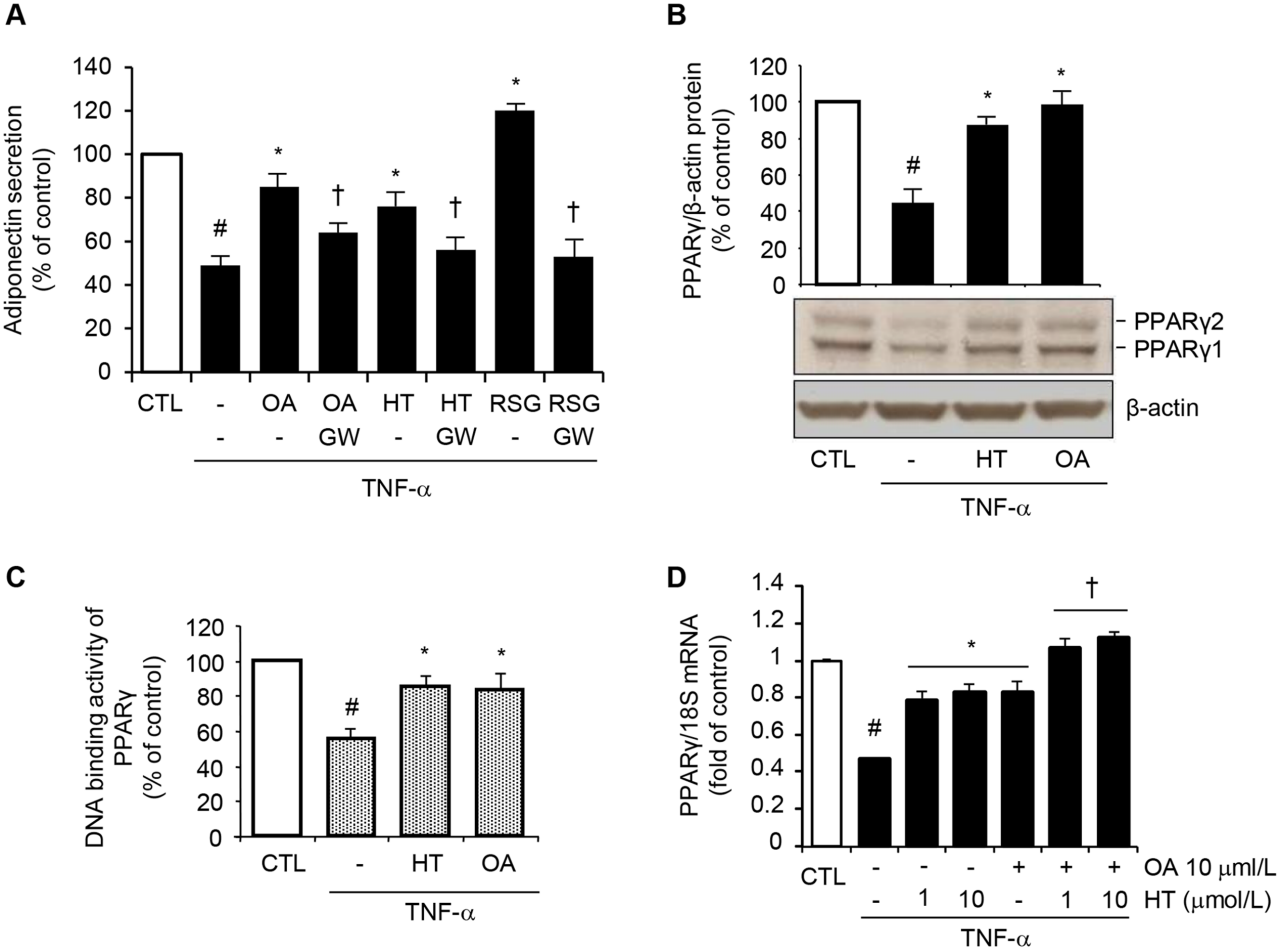
Attenuation by HT and OA of TNF-α-induced inhibition of PPARγ expression and activity. (A) SGBS cells were treated with 1 μmol/L HT, 10 μmol/L OA, or 1 μmol/L RSG in the absence or presence of the PPARγ antagonist GW9662 at 10 μmol/L (GW), and then stimulated with 10 ng/mL TNF-α for 24 h. Adiponectin levels in the culture medium were determined by ELISA, and expressed as percent of unstimulated control (CTL). Data are means ± SD (n = 3). #*p*<0.05 versus CTL. *p<0.05 versus TNF-α alone. †*p*<0.05 versus the compound-treated group without GW9662. (B) and (C) SGBS cells were treated with 1 μmol/L HT or 10 μmol/L OA before 10 ng/mL TNF-α stimulation for 24 h. (B) Whole-cell lysates were assayed by Western blotting using antibodies against PPARγ1, PPARγ2, and against β-actin, this last used as a loading control. Total PPARγ1 and PPARγ2 band intensities were normalized to β-actin, and are expressed as percent of unstimulated control (CTL). (C) Nuclear proteins were analyzed for PPARγ DNA-binding activity by ELISA as described in Methods. Data are expressed as percent of unstimulated control (CTL). (D) SGBS cells were treated with 1–10 μmol/L HT, or 10 μmol/L OA, or co-treated with OA + HT before 10 ng/mL TNF-α stimulation for 24 h. PPARγ mRNA levels were determined by qPCR and normalized to 18S RNA. Data are expressed as fold induction over unstimulated control (CTL). Bars represent means ± SD (n = 3). #*p*<0.05 versus CTL. *p<0.05 versus TNF-α. †*p*<0.05 versus each compound + TNF-α.

To understand the molecular mechanism(s) responsible for the HT- and OA-mediated restoration of adiponectin expression in inflamed adipocytes, we first evaluated the involvement of PPARγ, a master regulator of adiponectin gene expression [21], in the adiponectin upregulation by HT and OA. The induction of adiponectin release by 1 μmol/L HT and 10 μmol/L OA, as well as by RSG, was significantly abolished in the presence of GW9662, a selective PPARγ antagonist (Fig 5A), showing that PPARγ is implicated in adiponectin regulation by both HT and OA.

In other experiments, HT or OA significantly reverted TNF-α-induced reduction of both PPARγ1 and PPARγ2 protein expression at Western analysis (Fig 5B), as well as PPARγ DNA binding activity in nuclear extracts (Fig 5C). This effect was accompanied by a concomitant HT- or OA-mediated attenuation of the TNF-α-induced inhibition of PPARγ mRNA levels (Fig 5D). Again, the combination of OA with HT restored PPARγ gene expression in an additive manner compared with treatments with both the here studied olive oil components in isolation (Fig 5D). Similar results on PPARγ expression were obtained in 3T3-L1 adipocytes (S4 Fig). These data suggested that HT and OA can prevent the TNF-α-mediated inhibition of adiponectin expression at least in part by counteracting TNF-α inhibitory effect on PPARγ expression and activation.

### JNK is required for TNF-α-induced suppression of adiponectin

**Fig 6:**
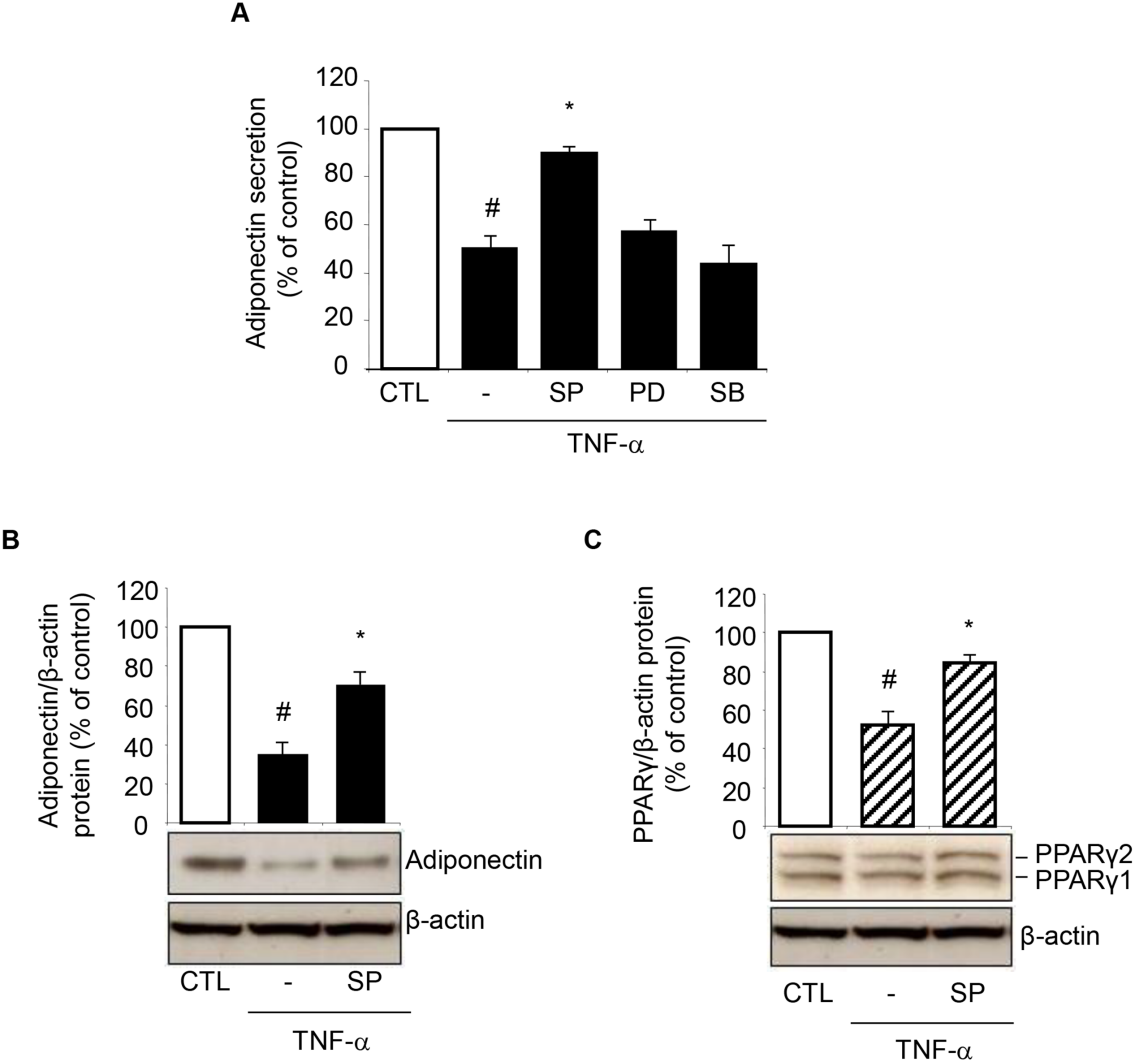
Involvement of JNK activation in TNF-α-induced PPARγ and adiponectin inhibition. SGBS cells were pretreated for 1 h with 10 μmol/L of the JNK inhibitor SP600125 (SP), the ERK1/2 inhibitor PD98059 (PD), or the p38 inhibitor SB203580 (SB), and then stimulated with 10 ng/mL TNF-α for 24 h. Culture media were analyzed for adiponectin by ELISA (A), and whole-cell lysates were assayed by Western blotting using antibodies against adiponectin (B) or PPARγ (C). Adiponectin and PPARγ expression were normalized to β-actin, and expressed as percent of unstimulated control (CTL). Bars represent means ± SD (n = 3). #*p*<0.05 versus CTL. *p<0.05 versus TNF-α.

In search of the upstream site of interference by HT and OA with the signaling pathway(s) mediating adiponectin and PPARγ suppression by TNF-α, and given the role of MAPK activation in adipocyte inflammatory responses [22], we examined the involvement of the MAPK JNK, extracellular signal-related kinase (ERK) and p38 in the regulation of adiponectin expression by TNF-α by using specific pharmacological MAPK inhibitors. Preliminary concentration-response study showed that concentrations of 10 μmol/L of the JNK inhibitor SP600125, the ERK1/2 inhibitor PD98059, and the p38 inhibitor SB203580 were effective in blunting TNF-α-induced phosphorylation of the specific target MAPK (not shown). We found that the JNK inhibitor SP600125, which inhibits the phosphorylation/activation of both JNK1 and JNK2 isoforms with similar potency [23], reverted the inhibition of adiponectin protein release in the cell culture medium by TNF-α, while PD98059 and SB203580 had no effect (Fig 6A). Therefore, it appeared that TNF-α inhibits adiponectin secretion at least in part by selectively activating JNK. Concordantly, JNK inhibition counteracted the reduction in the intracellular protein expression of adiponectin (Fig 6B), as well as PPARγ (Fig 6C) at Western analysis, suggesting the potential for JNK to be a molecular switch mediating the downregulation of PPARγ and the related adiponectin by TNF-α.

**Fig 7:**
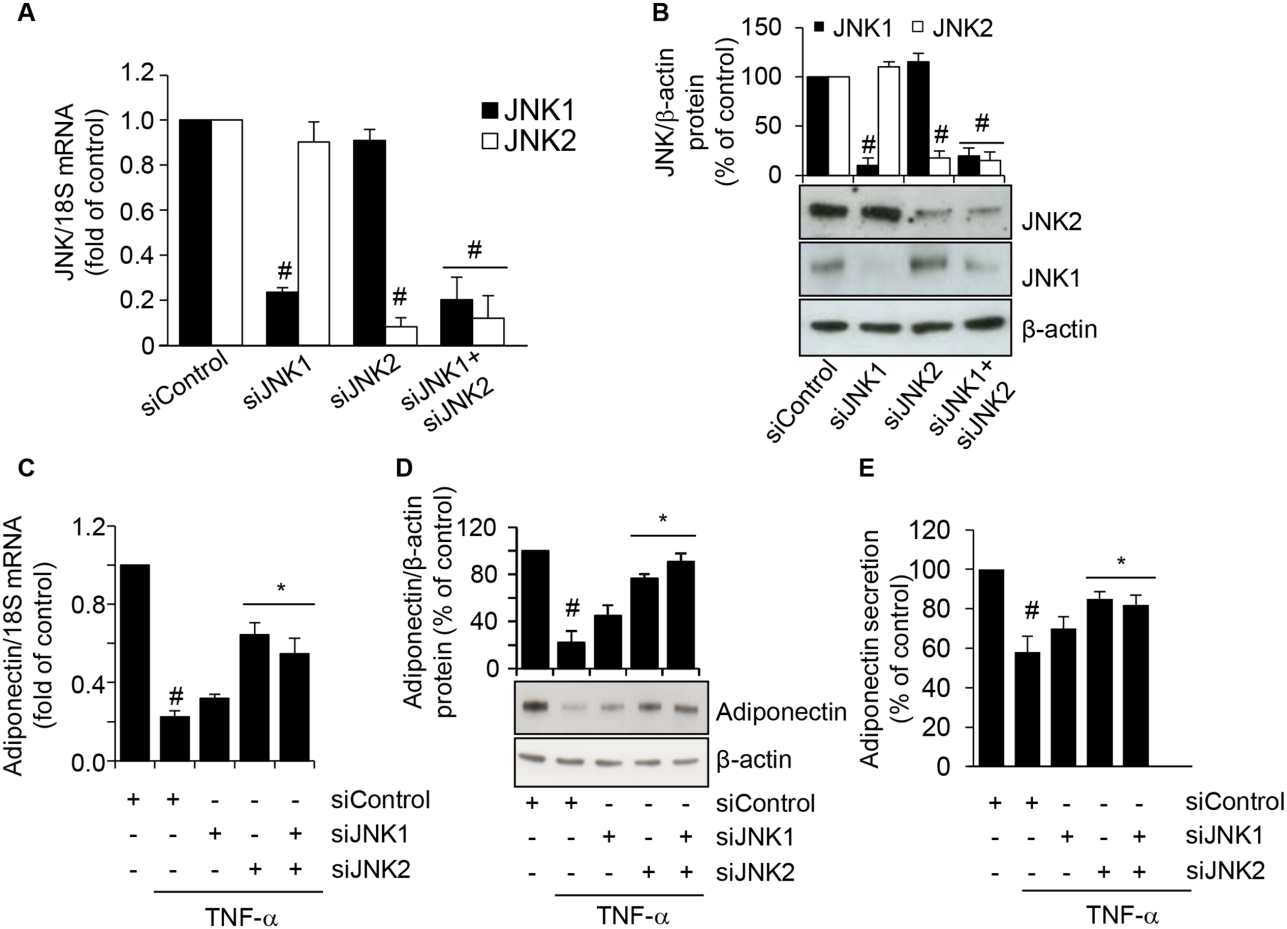
Attenuation of TNF-α-induced adiponectin downregulation by siRNA-mediated depletion of JNK. SGBS cells were treated with scrambled negative control siRNA (siControl), JNK1 siRNA (siJNK1), JNK2 siRNA (siJNK2), or JNK1 plus JNK2 siRNA, for 72 h. The mRNA expression levels of JNK1 and JNK2 were measured by qPCR, normalized to 18S RNA, and expressed as fold induction over scrambled negative control siRNA (A). JNK1 and JNK2 intracellular protein levels were assayed by Western blotting, normalized to β-actin, and expressed as percent of scrambled negative control siRNA (B). Bars represent means ± SD. #*p*<0.05 versus siControl. After 72 h of transfection, cells were stimulated with 10 ng/mL TNF-α for further 24 h. Adiponectin mRNA were determined by qPCR (C), while adiponectin intracellular and secreted protein levels were determined by Western analysis (D) and ELISA (E), respectively. Bars represent means ± SD. #*p*<0.05 versus siControl without TNF-α. *p<0.05 versus siControl with TNF-α.

To further verify the involvement of JNK in TNF-α-induced inhibition of adiponectin expression, and to ascertain the relative contribution of each JNK isoform, we transfected SGBS cells with either targeted JNK1 and/or JNK2 siRNA, and then stimulated them with TNF-α for 24 h. As shown in Fig 7A and 7B, compared with transfections with a scrambled negative control siRNA, siRNA-mediated knockdown of JNK1 or JNK2 resulted in a specific and significant reduction in the levels of the targeted JNK1 and JNK2 mRNAs and intracellular protein levels, respectively. We next examined the effect of JNK silencing on the TNF-α-induced downregulation of adiponectin expression. While JNK1 siRNA alone had no significant effect on adiponectin mRNA, intracellular and secreted protein levels, JNK2 siRNA or co-treatment with JNK1 and JNK2 siRNA significantly reversed the TNF-α-induced reduction of adiponectin expression and secretion (Fig 7C–7E), thus confirming that a JNK-, and, in particular, a JNK2-dependent pathway is required for the TNF-α-induced suppression of adiponectin.

### HT and OA additively reduce TNF-α-induced activation of JNK

**Fig 8:**
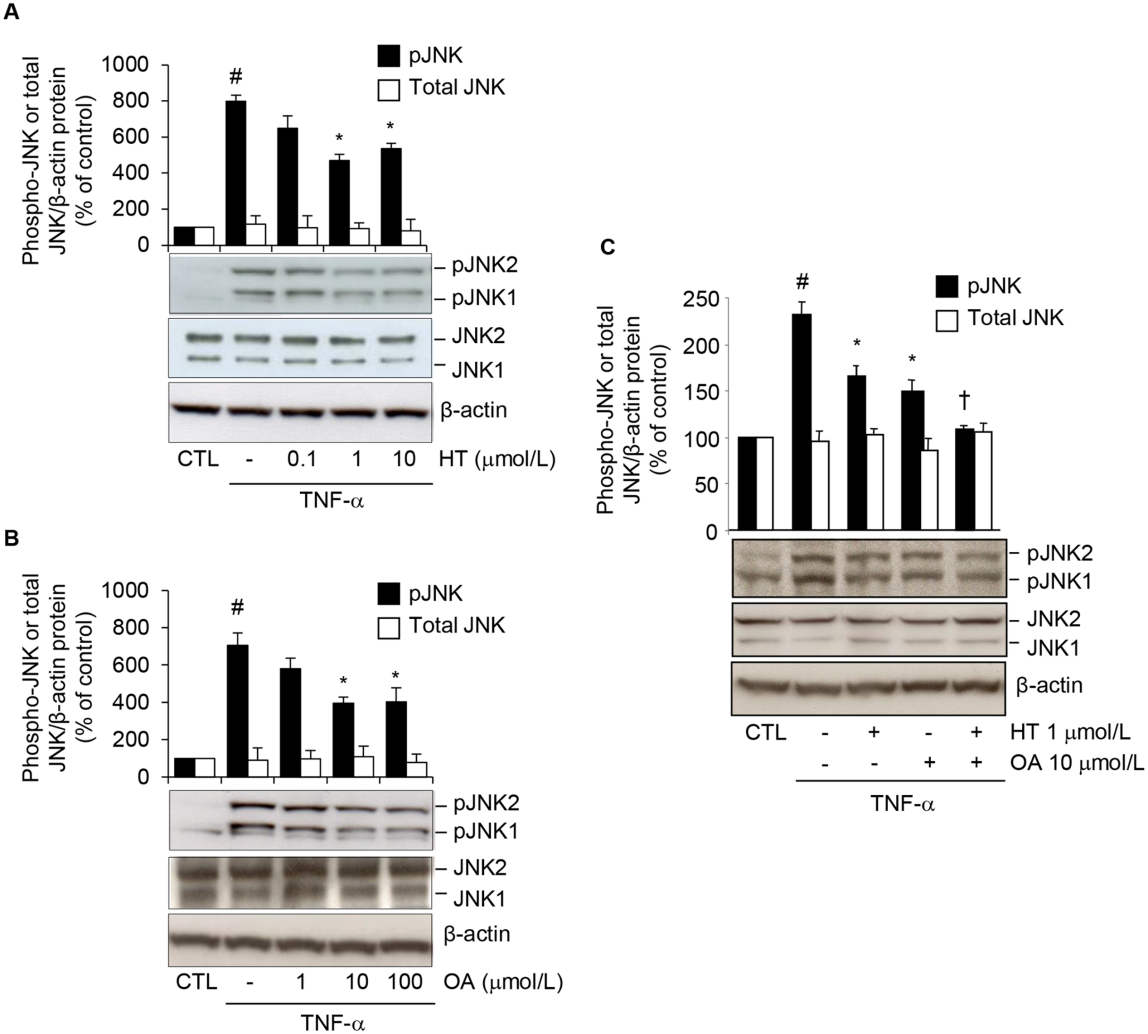
Attenuation by HT and OA of TNF-α-induced JNK phosphorylation. SGBS cells were treated with increasing concentrations of HT (A) or OA (B), or with 1 μmol/L HT, 10 μmol/L OA or co-treated with OA + HT (C) before 10 ng/mL TNF-α for 20 min. Whole-cell lysates were assayed by Western blotting using antibodies against phosphorylated (p) JNK1/2, total JNK or β-actin, as a loading control. Band intensities for phosphorylated and total JNK were normalized to β-actin, and are expressed as percent of unstimulated control (CTL). Bars represent means ± SD. #*p*<0.05 versus CTL. *p<0.05 versus TNF-α. †*p*<0.05 versus each compound + TNF-α.

To address the possibility of an interference of HT or OA with JNK activation, we evaluated HT or OA effects on JNK phosphorylation in response to TNF-α in human adipocytes. At Western analysis, HT and OA significantly and concentration-dependently reduced the TNF-α-induced increase of phosphorylated (p) JNK1 and JNK2 (Fig 8A and 8B), and this occurred in an additive manner (reduction by about 30 ± 4% for single treatments, and by 55 ± 2% for OA plus HT treatment, compared with TNF-α alone) (Fig 8C). Similar results were obtained in 3T3-L1 adipocytes (S4 Fig).

## Discussion

Adiponectin is an adipocyte-specific secretory protein with direct anti-diabetic, anti-atherogenic and anti-inflammatory properties [3]. The increase in adiponectin expression and plasma levels with drugs already existing or with novel therapeutic strategies is therefore considered valuable in the prevention and treatment of obesity-related metabolic and cardiovascular diseases in humans. The present study aimed at characterizing the effects of two representative and predominant virgin olive oil components, the MUFA OA and the antioxidant phenol HT, on adiponectin production by adipocytes stimulated with a prototypic inflammatory stimulus. We here demonstrate, for the first time, that both OA and HT, alone and in combination, and in this latter case additively, prevent TNF-α-induced suppression of adiponectin expression via the attenuation of JNK-mediated PPARγ downregulation.

We chose to determine OA and HT effects on adiponectin in the well-characterized and widely used human SGBS adipocytes as a cell model system closely resembling human native adipocytes [19]. We studied OA and HT effects on adiponectin also in the murine 3T3-L1 cell line, a widely used model for the study of adipocyte biology. In an attempt to reproduce the dysmetabolic and pro-inflammatory milieu causally linked to adipocyte dysfunction, we examined the effects of HT and OA on cultured adipocytes stimulated with the proinflammatory cytokine TNF-α, which is elevated in obesity and T2DM and is known to reduce adiponectin expression and secretion [24]. The exposure of adipocytes to TNF-α, as it may occur upon infiltration of TNF-α-producing leukocytes—mainly monocytes/macrophages—into the adipose tissue in conditions of obesity, causes adipocyte dysfunction, resulting in the acquisition of a proinflammatory state, accompanied by adiponectin suppression [24]. To our knowledge, this is the first demonstration of the capacity of OA and HT to antagonize TNF-α-induced suppression of adiponectin mRNA expression and protein release in human and murine adipocytes, thus pointing to a novel anti-inflammatory action of these two dietary factors.

These effects occurred for both compounds at nutritionally relevant concentrations. In fact, HT effective concentrations were as low as 1 μmol/L, which is in the range of plasma concentrations of HT and its metabolites (0.01 to 10 μmol/L) attainable after dietary consumption of virgin olive oil [25]. OA is one of the most abundant free FA in human serum: its plasma concentrations, normally ranging from 1 to 50 μmol/L, are positively related to chronic olive oil consumption [26]. OA significantly inhibited adiponectin downregulation at 10 μmol/L in human SGBS adipocytes, thus supporting the physiological relevance of our findings. In mouse 3T3-L1 adipocytes, OA was effective in counteracting adiponectin downregulation in inflamed adipocytes at a higher concentration (100 μmol/L) than in human SGBS adipocytes, reflecting the impact of species differences between human and murine adipocytes. However, we were able to demonstrate a similar favorable regulation of adiponectin in inflamed condition in two adipocyte model systems, thus strengthening our observations and allowing us to reasonably generalize such findings.

Another notable observation of the present study is the additive favorable effect on adiponectin expression when OA and HT were given in combination, compared with single treatments. In addition to vegetable oils, OA can be found in animal fat, which is however also rich in (presumably less healthy) saturated FAs. Notably, our result suggests that the combination of OA and HT, as peculiarly occurring in virgin olive oil, may be important *in vivo*, and indicates that the bioactivity of the parental nutrient is better appreciated when the natural food matrix is maintained. This observation is likely key to understand the relevance of *in vitro* studies with isolated compounds with the *in vivo* activity of Mediterranean diets [5,11,13,27–29].

To unveil the potential molecular mechanism(s) responsible for adiponectin restoration by OA and HT, we investigated their effects on the nuclear receptor PPARγ, the master transcriptional regulator of adiponectin [21]. The observed attenuation of OA- and HT-mediated increase in adiponectin release by PPARγ antagonism suggests a PPARγ-dependent mechanism underlying OA and HT effects. It can be hypothesized that OA and HT might act 1) as direct PPARγ agonists; and/or 2) as modulators of the TNF-α-triggered inflammatory signaling cascade leading to PPARγ inhibition and the related adiponectin suppression. Regarding the first hypothesis, unsaturated FAs are known endogenous ligands for PPARs [30], and some polyphenols also exhibit direct PPARγ binding activities [31]. However, in our hands OA and HT did not significantly modify either PPARγ DNA binding activity (not shown) or adiponectin production in unstimulated, non-TNF-α-treated adipocytes (a condition characterized by elevated expression and activity of PPARγ). Therefore, we rather hypothesized that OA and HT interfere with the suppressive effect of TNF-α on PPARγ expression levels and/or activity. This was confirmed by the significant recovery we observed in PPARγ mRNA and protein expression, accompanied by increased PPARγ DNA binding activity, after single treatment with OA or HT, and in an additive manner after combined OA + HT treatment. Therefore, both OA and HT likely attenuate TNF-α–mediated suppression of adiponectin, at least in part, by restoring PPARγ expression and activity.

Potential mechanisms by which OA and HT block TNF-α-induced cellular responses in adipocytes include the interference with receptor and/or post-receptor signaling pathways. It is known that TNF-α causes inflammation by triggering a cascade of serine/threonine kinase phosphorylations that activates MAPKs and transcription factors, such as nuclear factor (NF)-κB and activator protein (AP)-1. These factors act in concert to cause adipocyte dysfunction and insulin resistance directly by decreasing insulin signaling, or indirectly by inducing inflammatory gene expression and interfering with PPARγ activity [32]. Consistent with earlier studies [33,34], we found that JNK is an obligate mediator of the TNF-α-induced downregulation of both PPARγ and adiponectin in human adipocytes, since specific JNK pharmacological or molecular (siRNA) inhibition blunted the TNF-α suppressive effect on these two proteins. The JNK subgroup of MAPK is encoded by 3 genes. Of these, JNK1 and JNK2 are ubiquitously expressed and play important role in metabolic diseases [35,36]; JNK3 is expressed predominantly in the heart, testis and brain [37]. Interestingly, we found that siRNA-mediated depletion of JNK2, but not JNK1, significantly attenuated TNF-α-induced suppression of adiponectin, indicating for the first time a role for JNK2-mediated signaling in adiponectin expression in inflammatory condition. Although previous studies have reported that JNK1—but not JNK2—deficiency results in reduced adiposity and insulin resistance in mouse models of obesity [35], a role for JNK2 in metabolic regulation [36,38], as well as in atherogenesis [39], has been also appreciated. We here demonstrate for the first time that OA and HT attenuate TNF-α-mediated JNK1 and JNK2 activation, pointing to JNK as a mediator of OA- and HT-induced improvement in adiponectin expression and PPARγ activity. The similarity of effects of OA and HT on these early regulatory steps in adipocyte activation after TNF-α fully accounts for the additive—and not synergistic—nature of the effects described for the two compounds.

JNK activation is an emerging key element in the signaling pathway linking inflammation and metabolism, and represents a potential therapeutic target in obesity and T2DM [22]. JNK is activated by various stress signals, including cytokines and FFAs, leading to inflammatory responses as well as to insulin resistance [22]. Although previous studies have demonstrated JNK modulation by dietary compounds—including HT and OA—in other cellular models [40–42], ours is the first report of JNK inhibition by OA and HT in adipocytes, with a potential beneficial impact on obesity-related adipocyte dysfunction.

Further studies are, however, still warranted to precisely identify the upstream molecular target responsible for OA and HT inhibition of JNK. A potential mechanism by which OA and HT prevent adiponectin downregulation by TNF-α is the inhibition of TNF-α-induced production of intracellular reactive oxygen species (ROS) [43]. Increased ROS levels are documented in the adipose tissue of obese, insulin-resistant mice and humans, and are important triggers for inflammation and insulin resistance, being also able to negatively affect adiponectin release [43,44]. Interestingly, oxidative stress both activates JNK and is itself induced by JNK activation [43], and has been demonstrated to interfere with adipocyte PPARγ expression [44]. Since antioxidant properties have been attributed both to HT [13,45] and OA [27], and are functionally associated with a significant quenching of inflammatory responses, we infer that an antioxidant mechanism is a probable contributor to the observed improvement in adiponectin expression by both compounds. This hypothesis is under current investigation. Whether the positive effect of OA and HT on adiponectin is accounted for by the inhibition of other TNF-α-triggered intracellular pathways relevant to obesity (such as ceramide biosynthesis, endoplasmic reticulum stress, and mitochondrial dysfunction), also deserves further evaluation.

Our data on adiponectin regulation by OA further expand recent reports showing a significant increase in basal adiponectin expression in OA-treated 3T3-L1 adipocytes, and a positive association between serum adiponectin levels and OA content in the rat adipose tissue [29]. Additionally, the OA capacity to improve adiponectin expression in adipocytes may contribute to explaining the beneficial effects of OA-enriched diets on human adiponectin plasma levels [46,47], as well as on inflammation and insulin sensitivity [18,48,49]. The direct effect of OA on adiponectin may support the gene-diet interaction recently found between dietary MUFAs and an adiponectin gene polymorphism modulating body mass index and the risk of obesity [50]. On the other hand, HT has been also shown to inhibit preadipocyte differentiation [51], and to promote mitochondrial function in differentiated 3T3-L1 adipocytes [28] and in obese mice [17], thus suggesting protective effects against the development of obesity. Recent lines of evidence have also reported improvement of insulin sensitivity after supplementation of HT-rich extracts from olive leaves in overweight men [16], and beneficial effects against the development of the metabolic syndrome in animal models [17]. The present report of HT-mediated restoration of adiponectin gene expression in inflammatory conditions improves our understanding and prompts to further exploitation of the biological properties of olive oil phenols.

To substantiate our observations on favorable modification of adipocytes (dys)function by HT and OA, we determined their effect on the release of leptin, another major adipose tissue-derived adipokine. In addition to serve as an adipose signal for the long-term regulation of food intake, energy expenditure and body weight, leptin may contribute to the metabolic and vascular risk associated with obesity [4]. Unlike adiponectin, plasma leptin levels are increased in obesity and type 2 diabetes, reflecting a state of leptin resistance; and are independently associated with hypoadiponectinemia, insulin resistance, dyslipidemia, inflammatory markers, and CVD [4]. Moreover, since leptin and adiponectin are associated with opposite metabolic and vascular effects and contribute, usually in an opposite manner, to several components of the metabolic syndrome, the leptin to adiponectin ratio has been shown to reflect compromised adipose tissue function and to be a better predictor of insulin resistance and adverse outcomes, including CVD and mortality, than either leptin or adiponectin alone [52–54]. Therefore, we also assessed the role of HT and OA on the leptin to adiponectin ratio in adipocytes. We found that, while adiponectin remained unchanged, leptin release was significantly decreased in HT- or OA-treated cells compared with untreated control. As a consequence, the leptin-to-adiponectin ratio significantly decreased after HT or OA treatment in basal unstimulated control. In TNF-α-treated cells, in addition to adiponectin, leptin levels were also reduced, in agreement with previous studies [55,56]; HT or OA, however, while restoring adiponectin levels, did not affect TNF-α-stimulated reduction of leptin, a finding that suggests that TNF-α and HT or OA regulate leptin production through different mechanisms. The combined regulation of adiponectin and leptin by HT and OA upon TNF-α stimulation resulted in a further reduction of the leptin-to-adiponectin ratio. Further investigations, beyond the scope of the present study, are warranted to establish the mechanism(s) underlying leptin regulation by HT and OA, including the possible involvement of PPARγ or other yet unidentified signaling pathways. However, these results point to a beneficial effect by the olive oil components on adipokine profile, and complement other reports on the modulation of leptin production by HT and OA [45,57,58]. Moreover, our *in vitro* data on a favorable modification of the leptin-to-adiponectin ratio in adipocytes may corroborate some *in vivo* observations reporting an improvement of the leptin to adiponectin ratio, as well as other parameters related to glycemic control, by a Mediterranean diet supplemented with virgin olive oil in patients with type 2 diabetes [9].

**Fig 9:**
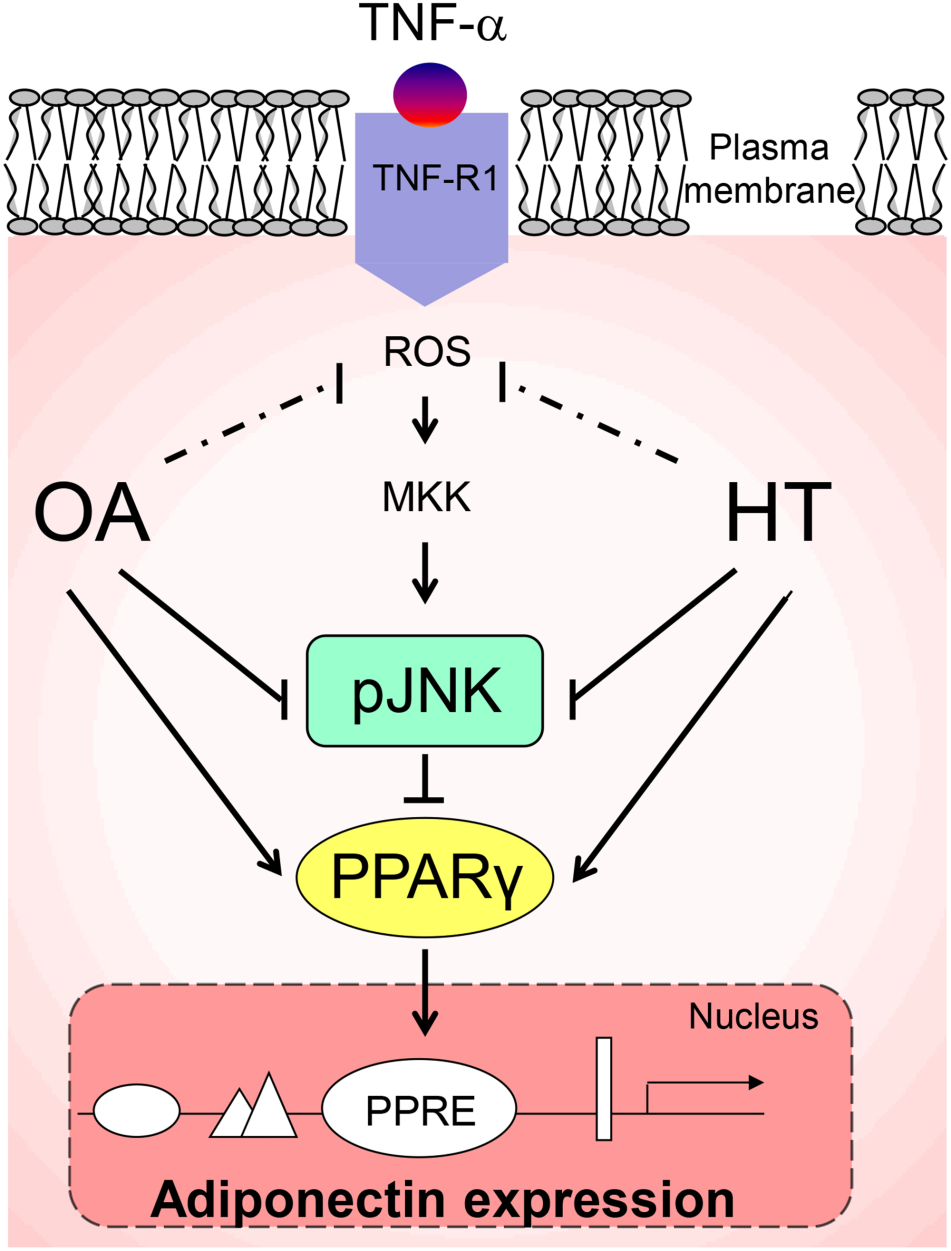
A working model for the HT and OA-mediated increase of adiponectin expression in TNF-α-stimulated adipocytes. Pre-treatment with HT and OA before TNF-α stimulation prevents JNK activation and restores PPARγ expression and activity and, as a consequence, adiponectin levels. A coherent interpretation of the findings is as follows: upon binding to the cognate receptor, TNF-α induces reactive oxygen species (ROS) production and triggers an inflammatory signaling cascade involving, among others, the activation of JNK, which mediates the degradation of PPARγ (a transcription factor implicated in adiponectin gene expression through a PPAR-responsive element (PPRE) in its promoter). As a result, adiponectin expression is downregulated. Arrow indicates stimulation. Line indicates inhibition. MKK: MAP kinase kinase; pJNK: phosphorylated JNK.

This is an i*n vitro* study, with all limitations in the cellular model here used for *in vivo* inferences. Possible specific limitations include the fact that we did not determine the effect of HT main metabolites (e.g., ortho-methylic derivatives and glucuronides) formed after olive oil ingestion and detected in human biological fluids along with the free form of HT; and the lack of data on adiponectin oligomers, critical in determining adiponectin systemic actions. Our current research is currently focusing on these important aspects. Strengths of our study however include the use of dietarily relevant concentrations of OA and HT; the simultaneous evaluation of the two most representative and chemically different components of virgin olive oil; and, possibly even more interesting, the demonstration of additive effects of HT and OA treatments. Indeed, testing the joint effects of bioactive compounds reproduces the natural food matrix of virgin olive oil more closely than the use of single, isolated components, as done in previous studies. In conclusions, we found that physiological concentrations of two virgin olive oil components, OA and HT, additively prevented inflammation-induced impairment of adiponectin in human adipocytes. Both compounds appear to exert their beneficial effects by decreasing TNF-α-induced JNK activation and improving PPARγ expression. A model, based on our data, of the mechanism by which OA and HT prevent TNF-α-induced impairment in adiponectin expression is shown in Fig 9. These results, in association with favorable changes of the leptin-to-adiponectin ratio, contribute to explaining the metabolic and cardiovascular protection provided by olive oil consumption in the context of the traditional Mediterranean diet.

## Supporting Information

S1 Fig Attenuation by HT and OA of TNF-α-induced inhibition of adiponectin protein release in mouse 3T3-L1 adipocytes. 3T3-L1 adipocytes were pretreated with HT (1 h) (A), OA (48 h) or RSG (24 h) (B) at the concentrations indicated, and then either treated with 10 ng/mL TNF-α, as a stimulus for adipocyte activation (filled bars), or left untreated (open bars), for 24 h. Adiponectin in the culture medium were determined by ELISA, and expressed as percent of unstimulated control (CTL). Bars represent mean ± SD (n = 3). #*p*<0.05 versus CTL. *p<0.05 versus TNF-α. **p<0.01 versus TNF-α.
(TIFF)

S2 Fig Attenuation by HT and OA of TNF-α-induced inhibition of adiponectin expression. 3T3-L1 adipocytes were pretreated with either HT, OA or cotreated with HT + OA before stimulation with TNF-α 10 ng/mL for 24 h. (A) Adiponectin in the culture medium were determined by ELISA, and expressed as percent of unstimulated control (CTL). (B) Adiponectin mRNA levels were determined by qPCR and normalized to 18S RNA. Data are expressed as fold induction over unstimulated control (CTL). (C) After treatments, cells were fixed and immunostained for adiponectin protein as described in Methods (x40 magnification). Scale bar = 100 μm. Quantification of immunostained adiponectin is shown in (D), and expressed as percent of unstimulated control (CTL). Data are means ± SD (n = 3). #*p*<0.05 versus CTL. *p<0.05 versus TNF-α alone. †*p*<0.05 versus each compound + TNF-α.
(TIFF)

S3 Fig The effect of HT and OA on leptin protein release and the leptin-to-adiponectin ratio. (A) SGBS adipocytes were pretreated with HT (1 h) or OA (48 h) at the concentrations indicated, and then either treated with 10 ng/mL TNF-α, or left untreated for 24 h. Leptin levels in the culture medium were determined by ELISA, and expressed as percent of unstimulated control (CTL). (B) The leptin-to-adiponectin ratio was calculated by dividing leptin by adiponectin concentrations, as determined by ELISA. Bars represent means ± SD (n = 4). #*p*<0.05 versus CTL. *p<0.05 versus TNF-α. †*p*<0.05 versus each compound + TNF-α.
(TIFF)

S4 Fig The effect of HT and OA on PPARγ expression and JNK phosphorylation in response to TNF-α. 3T3-L1 adipocytes were treated with HT, OA or co-treated with OA + HT at the concentrations indicated before 10 ng/mL TNF-α stimulation for 24 h (A), or for 20 min (B). (A) Whole-cell lysates were assayed by Western blotting using antibodies against PPARγ1, PPARγ2, and β-actin as a loading control. Total PPARγ1 and PPARγ2 band intensities were normalized to β-actin, and are expressed as percent of unstimulated control (CTL). (B) Whole-cell lysates were assayed by Western blotting using antibodies against phosphorylated (p) JNK, total JNK or β-actin, as a loading control. Phosphorylated and total JNK band intensities were normalized to β-actin, and are expressed as percent of unstimulated control (CTL). Bar represent mean ± SD. #*p*<0.05 versus CTL. *p<0.05 versus TNF-α. †*p*<0.05 versus each compound + TNF-α.
(TIFF)
