# Supplementary figures and images for: Additive Regulation of Adiponectin Expression by the Mediterranean Diet Olive Oil Components Oleic Acid and Hydroxytyrosol in Human Adipocytes
Source: PLoS One. 2015 Jun 1;10(6):e0128218. doi: 10.1371/journal.pone.0128218 (PMC4452359; doi:10.1371/journal.pone.0128218)

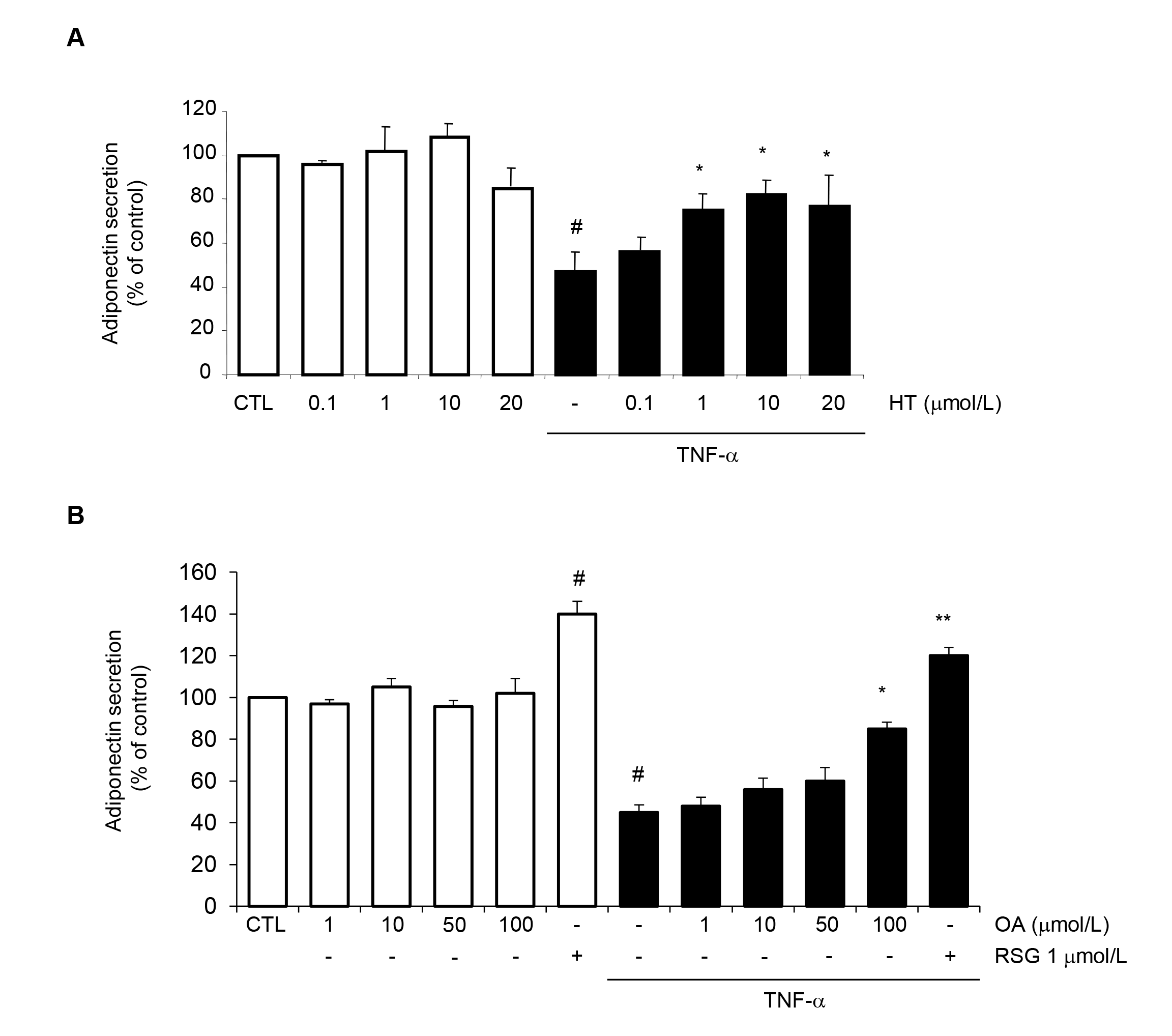

Supplement: S1 Fig — 3T3-L1 adipocytes were pretreated with HT (1 h) (A), OA (48 h) or RSG (24 h) (B) at the concentrations indicated, and then either treated with 10 ng/mL TNF-α, as a stimulus for adipocyte activation (filled bars), or left untreated (open bars), for 24 h. Adiponectin in the culture medium were determined by ELISA, and expressed as percent of unstimulated control (CTL). Bars represent mean ± SD (n = 3). #p<0.05 versus CTL. *p<0.05 versus TNF-α. **p<0.01 versus TNF-α. (TIFF) [file pone.0128218.s001.tiff]

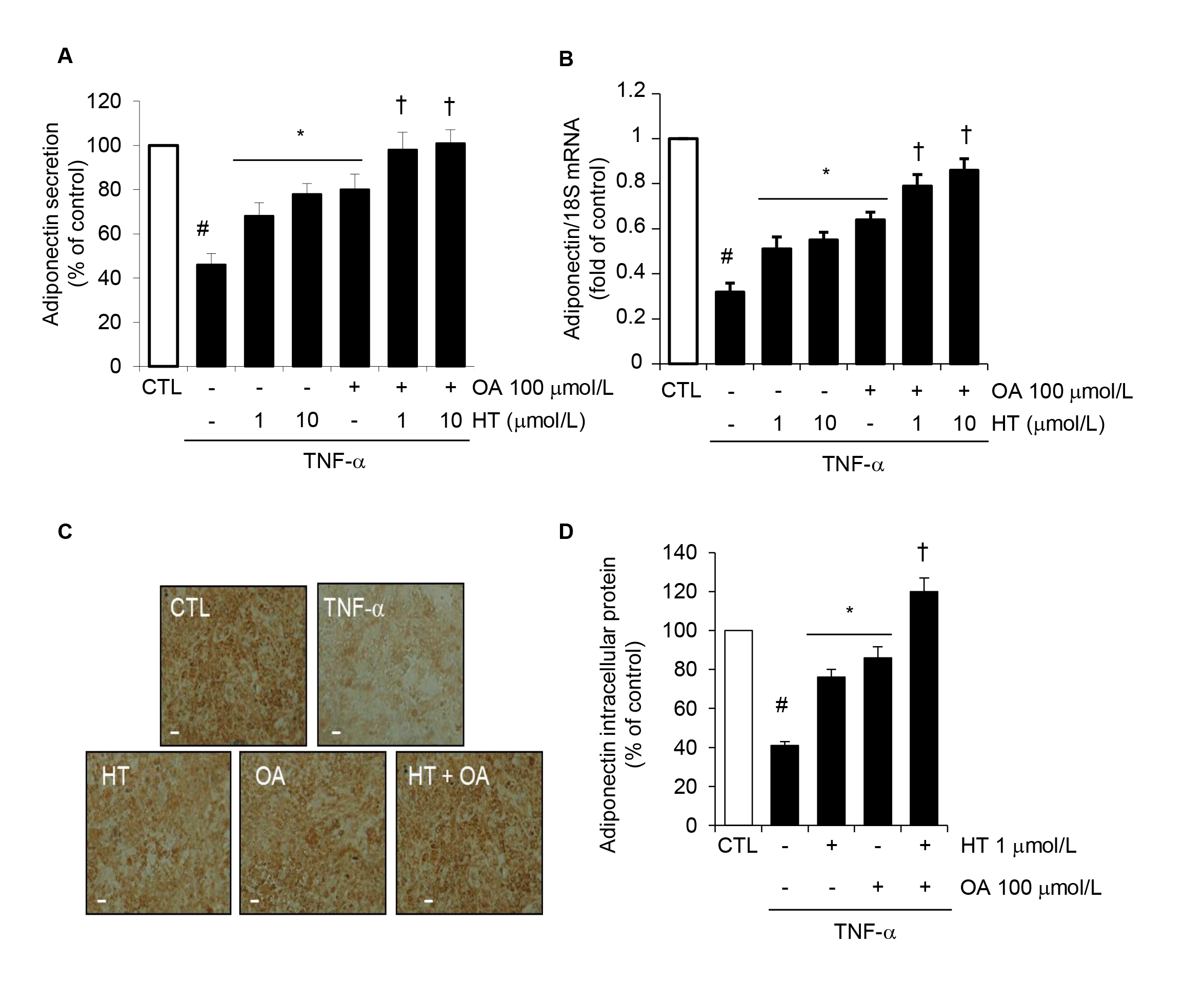

Supplement: S2 Fig — 3T3-L1 adipocytes were pretreated with either HT, OA or cotreated with HT + OA before stimulation with TNF-α 10 ng/mL for 24 h. (A) Adiponectin in the culture medium were determined by ELISA, and expressed as percent of unstimulated control (CTL). (B) Adiponectin mRNA levels were determined by qPCR and normalized to 18S RNA. Data are expressed as fold induction over unstimulated control (CTL). (C) After treatments, cells were fixed and immunostained for adiponectin protein as described in Methods (x40 magnification). Scale bar = 100 μm. Quantification of immunostained adiponectin is shown in (D), and expressed as percent of unstimulated control (CTL). Data are means ± SD (n = 3). #p<0.05 versus CTL. *p<0.05 versus TNF-α alone. †p<0.05 versus each compound + TNF-α. (TIFF) [file pone.0128218.s002.tiff]

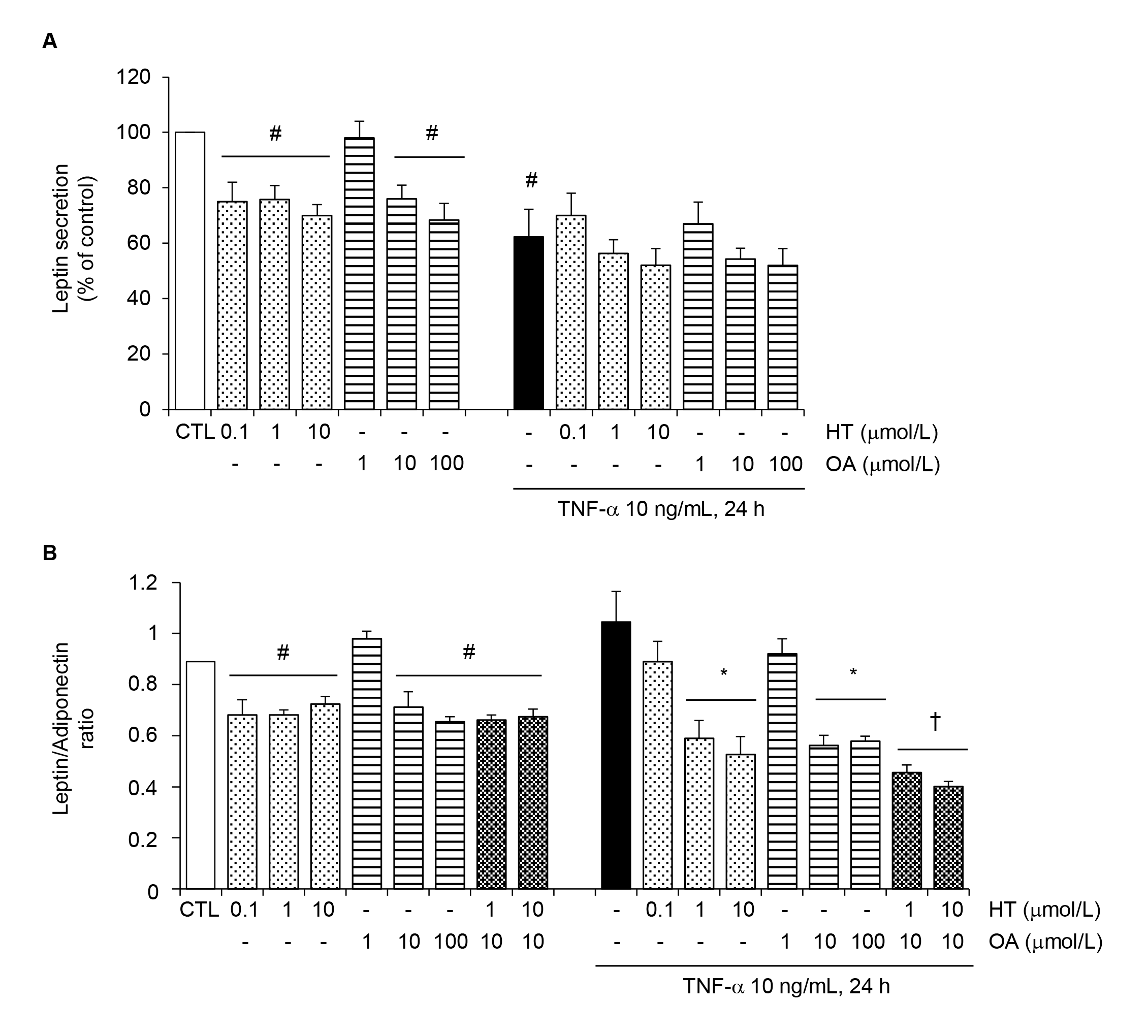

Supplement: S3 Fig — (A) SGBS adipocytes were pretreated with HT (1 h) or OA (48 h) at the concentrations indicated, and then either treated with 10 ng/mL TNF-α, or left untreated for 24 h. Leptin levels in the culture medium were determined by ELISA, and expressed as percent of unstimulated control (CTL). (B) The leptin-to-adiponectin ratio was calculated by dividing leptin by adiponectin concentrations, as determined by ELISA. Bars represent means ± SD (n = 4). #p<0.05 versus CTL. *p<0.05 versus TNF-α. †p<0.05 versus each compound + TNF-α. (TIFF) [file pone.0128218.s003.tiff]

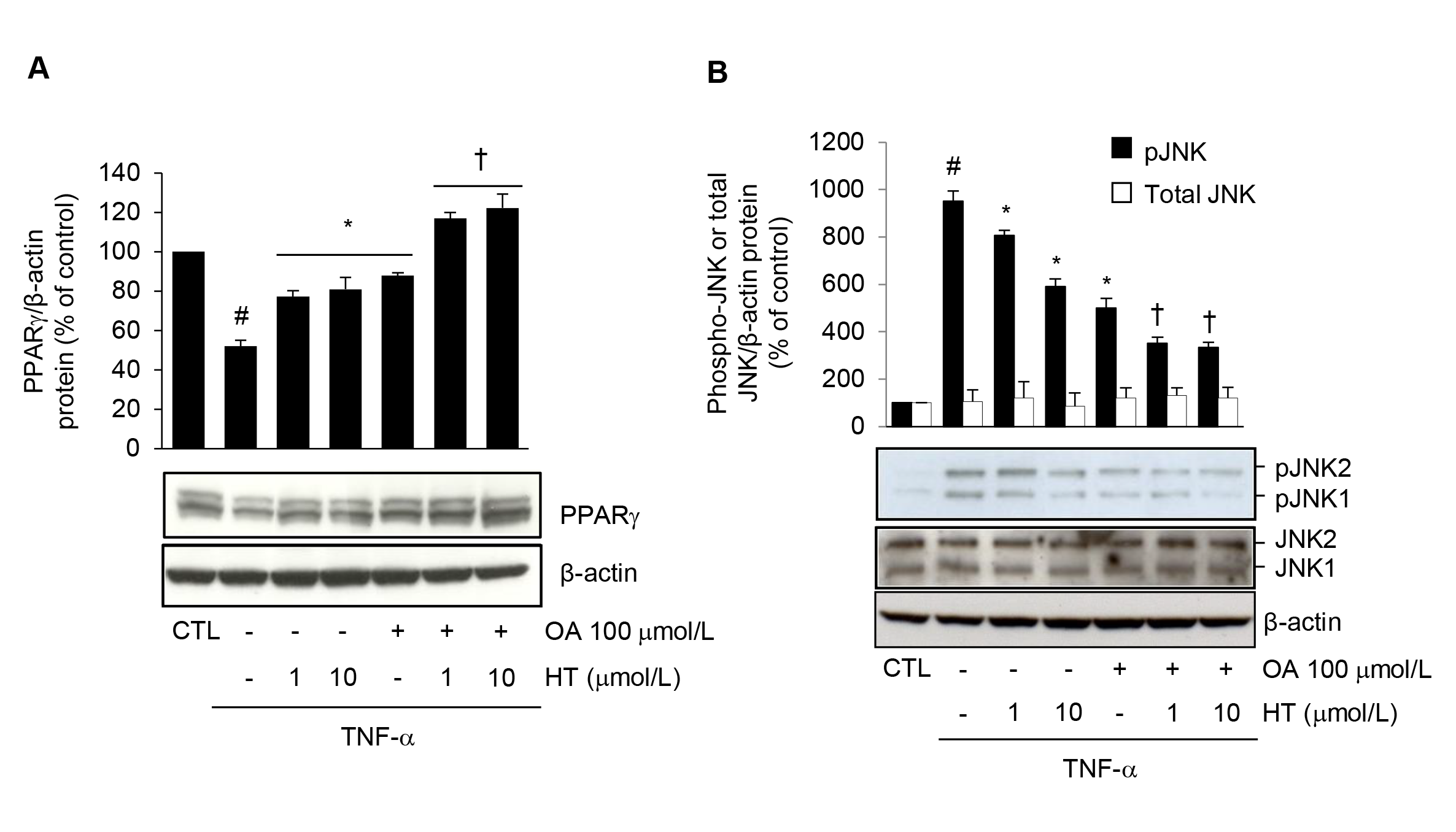

Supplement: S4 Fig — 3T3-L1 adipocytes were treated with HT, OA or co-treated with OA + HT at the concentrations indicated before 10 ng/mL TNF-α stimulation for 24 h (A), or for 20 min (B). (A) Whole-cell lysates were assayed by Western blotting using antibodies against PPARγ1, PPARγ2, and β-actin as a loading control. Total PPARγ1 and PPARγ2 band intensities were normalized to β-actin, and are expressed as percent of unstimulated control (CTL). (B) Whole-cell lysates were assayed by Western blotting using antibodies against phosphorylated (p) JNK, total JNK or β-actin, as a loading control. Phosphorylated and total JNK band intensities were normalized to β-actin, and are expressed as percent of unstimulated control (CTL). Bar represent mean ± SD. #p<0.05 versus CTL. *p<0.05 versus TNF-α. †p<0.05 versus each compound + TNF-α. (TIFF) [file pone.0128218.s004.tiff]
